# Fine Characterisation of a Recombination Hotspot at the *DPY19L2* Locus and Resolution of the Paradoxical Excess of Duplications over Deletions in the General Population

**DOI:** 10.1371/journal.pgen.1003363

**Published:** 2013-03-21

**Authors:** Charles Coutton, Farid Abada, Thomas Karaouzene, Damien Sanlaville, Véronique Satre, Joël Lunardi, Pierre-Simon Jouk, Christophe Arnoult, Nicolas Thierry-Mieg, Pierre F. Ray

**Affiliations:** 1 Equipe “Génétique, Infertilité et Thérapeutiques,” Laboratoire AGIM, CNRS FRE3405, Université Joseph Fourier, Grenoble, France; 2 Biochimie et Génétique Moléculaire, DBTP, CHU Grenoble, Grenoble, France; 3 Departement de Génétique et Procréation, Hôpital Couple Enfant, CHU de Grenoble, Grenoble, France; 4 Hospices Civils de Lyon, Centre de Biologie et de Pathologie Est, Service de Cytogénétique Constitutionnelle, Lyon, France; 5 Université Joseph Fourier, Centre National de la Recherche Scientifique, Laboratoire TIMC-IMAG UMR 5525, Grenoble, France; University of Pennsylvania, United States of America

## Abstract

We demonstrated previously that 75% of infertile men with round, acrosomeless spermatozoa (globozoospermia) had a homozygous 200-Kb deletion removing the totality of DPY19L2. We showed that this deletion occurred by Non-Allelic Homologous Recombination (NAHR) between two homologous 28-Kb Low Copy Repeats (LCRs) located on each side of the gene. The accepted NAHR model predicts that inter-chromatid and inter-chromosome NAHR create a deleted and a duplicated recombined allele, while intra-chromatid events only generate deletions. Therefore more deletions are expected to be produced de novo. Surprisingly, array CGH data show that, in the general population, DPY19L2 duplicated alleles are approximately three times as frequent as deleted alleles. In order to shed light on this paradox, we developed a sperm-based assay to measure the de novo rates of deletions and duplications at this locus. As predicted by the NAHR model, we identified an excess of de novo deletions over duplications. We calculated that the excess of de novo deletion was compensated by evolutionary loss, whereas duplications, not subjected to selection, increased gradually. Purifying selection against sterile, homozygous deleted men may be sufficient for this compensation, but heterozygously deleted men might also suffer a small fitness penalty. The recombined alleles were sequenced to pinpoint the localisation of the breakpoints. We analysed a total of 15 homozygous deleted patients and 17 heterozygous individuals carrying either a deletion (n = 4) or a duplication (n = 13). All but two alleles fell within a 1.2-Kb region central to the 28-Kb LCR, indicating that >90% of the NAHR took place in that region. We showed that a PRDM9 13-mer recognition sequence is located right in the centre of that region. Our results therefore strengthen the link between this consensus sequence and the occurrence of NAHR.

## Author Summary

We demonstrated previously that most men with globozoospermia, who produce only round acrosomeless spermatozoa and are 100% infertile, had a homozygous deletion removing the totality of *DPY19L2*. We also showed that this deletion occurred by Non-Allelic Homologous Recombination (NAHR). NAHR results in the production of deletions and duplications of regions encompassed by two homologous sequences, normally with a higher occurrence of deletions over duplications. Analysis of public databases at the *DPY19L2* locus paradoxically revealed that, in the general population, duplications were approximately three times as frequent as deletions. Analysis of sperm DNA permits us to quantify *de novo* events that take place during male meiosis. We therefore measured the rates of *de novo* deletion and duplication in the sperm of three healthy donors. As predicted by the NAHR theoretical model and contrary to the allelic frequency observed in the general population, we identified an approximate 2-fold excess of deletions over duplications. We calculated that the measured rate of *de novo* deletion was compensated by evolutionary loss, whereas duplications, not subjected to selection, increased gradually. Purifying selection against infertile homozygous deleted men may be sufficient for this compensation, or heterozygously deleted men may also suffer a small fitness penalty.

## Introduction

**Figure 1:**
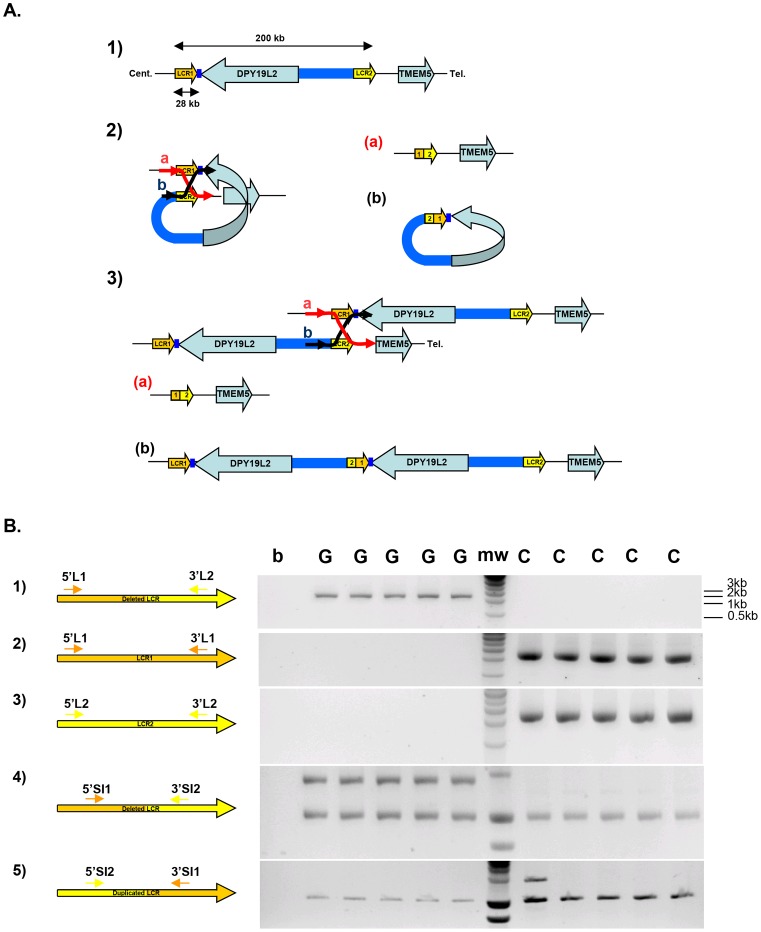
Strategy and validation of the detection of *DPY19L2* recombined alleles by PCR. (A) Schematic representation of NAHR at the *DPY19L2* locus. 1) LCR1 and LCR2 correspond to the centromeric and telomeric LCRs respectively. The two LCRs are separated by approximately 200 Kb and each measures 28 Kb. 2) NAHR can occur following the mis-alignment of Low Copy Repeats 1 and 2 located either on 1) the same chromatid and results in the production of a) a deleted allele with a recombined 1-2 LCR, and b) a small circular molecule with a recombined 2-1 LCR and the *DPY19L2* gene. This small molecule will not survive through the cell cycle. 3) NAHR can occur following the mis-alignment from two distinct chromatids (whether sister-chromatids or chromatids from homologous chromosomes). This results in the production of a) a deleted allele with a 1-2 recombined LCR, and b) a complementary duplicated allele with a 2-1 recombined LCR. (B) Illustration of the specificity of the LCR-specific amplification when amplifying DNA from *DPY19L2* homozygously deleted globozoospermic patients (G) and control individuals (C). 1) Primers specific to the deleted 1-2 LCR yield a 2088 nt fragment in globozoospermic patients only. 2,3) Specific amplification of LCR 1 and 2 is only obtained from non-deleted controls. 4) Co-amplification of a control locus (bottom band) with a deleted 1-2 LCR-specific sequence. 5) Co-amplification of a control locus (bottom band) with a duplicated 2-1 LCR-specific sequence. A duplicated allele is identified in one control individual (first lane after the molecular weight markers (mw)).

Several mechanisms have been proposed to cause genomic rearrangements, notably: Non Allelic Homologous Recombination (NAHR), Non Homologous End Joining (NHEJ), Fork Stalling and Template Switching (FoSTeS) and Break-Induced Replication (BIR) [1], [2]. NAHR takes place between duplicated sequences with a high sequence identity (usually >95%) located in different genomic regions of the same chromosome [3]. These paralogous sequences or Low Copy Repeats (LCR) tend to generate polymorphic regions with deleted and duplicated alleles called Copy Number Variants (CNVs). The consensual NAHR model predicts that recombinations between LCRs located on the same chromatid result in the production of a deleted allele and a small circular molecule that will be lost by the end of the cell cycle. Recombinations between LCRs located on two distinct chromatids (whether sister-chromatids or chromatids from homologous chromosomes) result in the production of a deleted allele and a complementary duplicated allele (Figure 1A). In consequence NAHR is expected to produce an excess of deletions over duplications. This has been verified for several NAHR hotspots using sperm typing assays: on average twice as many deletions as duplications were generated *de novo* [4]. One study however describes similar deletion and duplication frequencies at the 7q11.23, 15q11-q13 and 22q11.2 loci, suggesting a predominant inter-chromatid NAHR [5]. This study was carried out by fluorescent in situ hybridization (FISH) which allows the detection of all numerical anomalies occurring at these loci and not only the NAHR-mediated events. This could explain at least part of the discrepancy observed between the two studies, given that a recent study of the *RAI1* locus suggested that complex genetic events generate an excess of duplications [6].

As NAHRs events occur at fixed LCRs they tend to be recurrent, and the recombined alleles normally share a common size defined by the distance separating the two LCRs. It is well-established that meiotic recombination events, whether resulting in crossing over or producing unbalanced alleles through NAHR, are not uniformly distributed along the human genome but occur preferentially at specific hot spots [7]–[9]. Myers et al. (2008) have characterized a degenerate 13 bp sequence motif (CCNCCNTNNCCNC) that is present in approximately 40% of the identified human crossover hotspots. A three nucleotide periodicity was observed within and beyond the 13-mer core, suggesting a direct interaction with a motif binding protein [10]. Subsequent work strengthened this hypothesis as it has been proposed that PRDM9, a multi-unit zinc finger binding protein expressed mainly during early meiosis in germ cells [11], specifies hotspot usage by binding specifically to this 13 bp consensus motif [12]–[14]. *PRDM9* was then shown to be highly polymorphic, different alleles seemingly providing preferred targeted recombination hotspots [14]. Berg et al. (2010) measured the recombination rate at ten crossover hotspots, five with a *PRDM9* recognition motif, five without a clear motif. Men with the rarer N allele showed a heavy reduction (>30-fold) at all hotspots, even at those which did not contain an obvious *PRDM9* motif [12]. Further work revealed that specific *PRDM9* alleles activated different hotspots [15]. The direct correlation between *PRDM9* recognition sequence and PRDM9 genotype however remains elusive, indicating that the rules governing the interaction between *PRDM9* and its targeted sequences must be subtle and complex [12], [15].

CNVs and other unbalanced micro recombination events are involved in the aetiology of many human pathologies such as Alpha Thalassemia, Potocki-Lupski Syndrome, Charcot-Marie Tooth, Williams-Beuren syndrome, Prader Willi/Angelman syndrome, and infertility through the production of Y-chromosome microdeletions [16]. Here we focus on the *DPY19L2* locus (12q14.2) which has recently been shown to be linked with Globozoospermia [17], a rare syndrome of male infertility [18] characterized by the presence of 100% round, acrosomeless spermatozoa in the patient's ejaculate (MIM #102530). Reports of familial cases pointed to a genetic component to this pathology [19]–[21], and this assumption was confirmed as a homozygous mutation of *SPATA16* was identified in three siblings [22] and a homozygous missense mutation of *PICK1* was identified in a Chinese patient [23]. We demonstrated recently that *DPY19L2* was in fact the main locus associated with globozoospermia as 15 out of 20 analysed patients presented a 200 Kb homozygous deletion removing the totality of the gene [17]. *DPY19L2* was described to have arisen, along with three other genes (*DPY19L1*, *L3* and *L4*), through the expansion and evolution of the *DPY19L* gene family from a single ortholog found in invertebrate animals [24]. We then identified *DPY19L2* point mutations and heterozygous deletions and demonstrated that 84% of the 31 globozoospermia patients analysed had a molecular alteration of *DPY19L2* [25]. Others find a slightly lower incidence of *DPY19L2* deletions in globozoospermia patients [26], [27]. Comparison of the spermiogenesis between wild type and *Dpy19l2* knock out (KO) mice allowed us to demonstrate that *Dpy19l2* is expressed in the inner nuclear membrane only in the section facing the acrosome, and that it is necessary to anchor the acrosome to the nucleus. This indicates that DPY19 proteins (*DPY19L1-4* in mammals) might constitute a new family of structural transmembrane proteins of the nuclear envelope that likely participate in a function that was so far known to be only carried out by SUN proteins: constituting a bridge between the nucleoskeleton and cytoplasmic organelles and/or the cytoskeleton [28]. In our previous work we had demonstrated that *DPY19L2* was homozygously deleted in a majority of patients with globozoospermia and that this deletion occurred by NAHR between two highly homologous 28 Kb LCRs located on each side of the gene [17]. Strengthening the case for the occurrence of NAHR at the *DPY19L2* locus, heterozygous deletions and duplications have been identified in several large array CGH studies and this locus is classified as a CNV [29]–[33]. Surprisingly, considering that NAHR is known to generate an excess of deletions, these databases contain a large excess of duplications.

We developed a PCR assay to specifically amplify the recombined LCRs corresponding to deleted and duplicated alleles allowing the precise localisation of the breakpoints (BP). We observed that all identified BPs clustered in the center of the LCR. We analysed this region and identified a 13-mer PRDM9 pro-recombination sequences in the middle of the hotspot. We also developed a digital PCR assay that enabled us to estimate the rates of *de novo* deletion and duplication at this locus. Contrary to the allelic frequency observed in the general population we measured an approximate 2 fold excess of deletions over duplications. We show that the negative selection against the deleted alleles could explain this apparent paradox.

## Results

### Estimation of the *DPY19L2* deleted and duplicated alleles' frequencies in the general population and assessment of the PCR assay's sensitivity

The *DPY19L2* CNV was analysed using array CGH data available from web servers [29]–[33] for a total of 6575 control individuals, mainly from the Database of Genomic Variants (http://projects.tcag.ca/variation/). A total of 83 gains and 26 heterozygous losses are reported for the *DPY19L2* CNV in this pool, indicating a threefold excess of duplications over deletions.

We wanted to confirm this result and exclude a potential technical bias towards duplications that could be caused by the presence on chromosome 7 of *DPY19L2P1*, a pseudogene highly homologous to *DPY19L2* [24]. To this end we re-analysed the array CGH data produced for the diagnosis of syndromic mental retardation in Grenoble and Lyon hospitals, and searched for *DPY19L2* deleted and duplicated alleles in this dataset. A total of 1699 array CGH profiles were re-analysed (see Figure S1 for illustration). We identified a total of 15 duplications and 3 heterozygous deletions. The recombined alleles were secondarily amplified with the long PCR primers to confirm the validity of the array CGH results. Presence of the deletion could be confirmed by our deletion-specific PCR in the three individuals putatively carrying a heterozygous deletion. DNA from 3 individuals expected to carry a duplicated allele could not be obtained. Ten out of the 12 remaining individuals putatively carrying a *DPY19L2* duplication were amplified by our duplication-specific PCR. For the two individuals that could not be amplified, the duplication was nevertheless confirmed by Multiplex Ligation-dependent Probe Amplification (MLPA). Theses results show that our reanalysis of the array CGH data did not yield any false positives. Overall reanalysis of these 15 individuals showed that 2 out of 15 recombinant alleles could not be detected by our PCR assay, indicating that the breakpoints of 2/15 recombined alleles fell outside of our amplified region.

We also wanted to obtain an estimation of the frequency of the deleted and duplicated alleles in the general population using our recombination-specific PCR assay. For that we designed primers that amplified a smaller sequence which could be co-amplified with an additional pair of primers (RYR2 primers) used as a positive amplification control (Figure 1B and Table S1). This duplex PCR setup controls for poor DNA quality or technical variations. We analysed 150 control individuals originating from North Africa and 150 individuals of European origin with these two duplex PCRs (for the detection of deleted and duplicated LCRs, respectively). We identified only one heterozygous deletion in an individual of North African origin and two duplications in one European and in one North African individuals.

Overall a total of 8574 individuals have been analysed, including 6575 individuals from array CGH public databases, 1699 individuals from Grenoble-Lyon array CGH data and 300 individuals analysed by recombination-specific PCR. From these cohorts we identified 30 deletions (frequency of approximately 1/290) and 100 duplications (approximate frequency 1/85) (Table S2). These values indicate that the allele frequencies of the recombined deleted and duplicated alleles are 1.7×10^−3^ (95% CI: 1.2×10^−3^; 2.5×10^−3^) and 5.8×10^−3^ (95% CI: 4.7×10^−3^; 7.1×10^−3^), respectively. Confidence intervals (CI) were calculated assuming a binomial model, with binom.test in R.

We note that our PCR-based assay only allows the identification of breakpoints occurring between the selected primers (1392 bp). The location of the breakpoints of each CNV detected by array CGH (an unbiased approach) located in the *DPY19L2* locus was scrutinised to establish if they were located within the LCR and hence were caused by NAHR (Table S3). This analysis shows that 87% of the deletions and 76% of the duplication fell within the LCR limits.

Overall, we believe that our PCR assay permits to identify the majority of recombinations occurring at the *DPY19L2* locus, since: 1) amplification was obtained for all 15/15 globozoospermia patients analysed, and 2) amplification was obtained for 13/15 (87%) recombined array CGH patients.

### Determination of *DPY19L2 de novo* recombination rates by digital PCR

As the previous results consistently showed an excess of duplications over deletions in the general population, we wanted to measure the rates of *de novo* duplications and deletions to verify if the observed skew was due to the selection of duplications over deletions or if more duplications were produced *de novo*. The rate of genetic events occurring *de novo* can be measured on sperm DNA since each spermatozoon is the product of meiosis and corresponds to a new haploid genome. We first tried to develop a semi-quantitative PCR assay to directly measure the frequencies of deletions and duplications using sperm from control donors (with two copies of *DPY19L2*). The shortest fragment that could provide a reliable specific amplification and amplify the whole breakpoint area was 1392 nt long. Reliable quantitative PCR for fragments longer than 500 nt is difficult with current techniques. We therefore resorted to performing a digital PCR. First, the DNA was serially diluted and distributed in 96-well plates so that approximately 25% of the wells produced an amplicon. The appropriate quantity of sperm DNA was determined by trial experiments for each of the two PCR assays: 50 ng of sperm DNA per well (corresponding to approximately 17,000 copies of chromosome 12, assuming one haploid genome represents 3 pg of DNA) were used for the PCR specific of the *DPY19L2* deletion, and 100 ng per well (∼33,000 copies under the same assumption) were used for the duplication-specific PCR.

**Figure 2:**
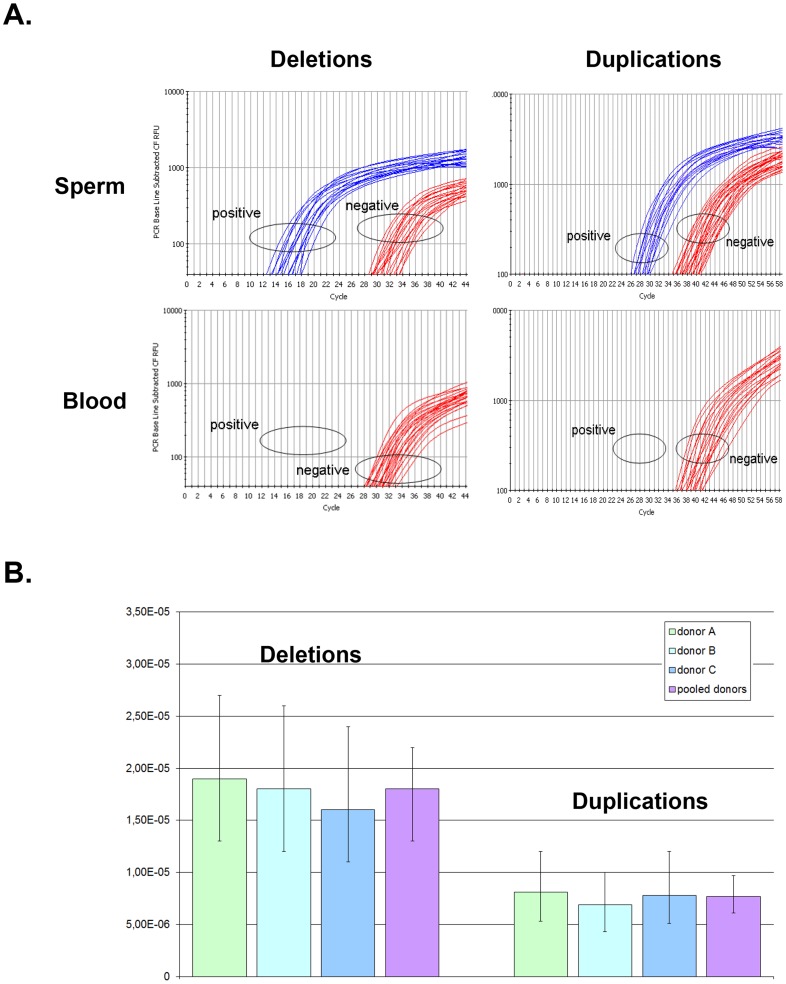
Rate of *de novo* deletion and duplication events occurring at the *DPY19L2* NAHR hotspot determined by digital PCR on sperm from 3 control donors. (A) Illustration of PCR results obtained by real time PCR. The left plots show amplification profiles obtained with primers specific to the recombined deleted LCR, the right plots show profiles obtained with the duplication-specific primers. No amplification was observed with either pairs of primers from 200 ng of somatic (blood) DNA, indicating that the NAHR did not occur during mitosis. Sperm DNA was diluted in order to obtain a positive amplification in approximately 25% of the wells. (B) The number of positive wells allowed estimating the frequency of *de novo* deletion and duplication events in three control sperms. Error bars represent 95% CIs.

For example, for donor A the deletion-specific PCR produced 26 positive wells. The deletion recombination frequency λ and its 95% confidence interval were then calculated as described (see Methods), resulting in a rate of *de novo DPY19L2* deletion for donor A estimated at 1.9×10^−5^ (95% CI: 1.3×10^−5^; 2.7×10^−5^). Similarly, the duplication-specific PCR for donor A produced 23 positive wells, but because there was twice as much starting DNA this results in a rate of *de novo DPY19L2* duplication estimated at 8.1×10^−6^ (95% CI: 5.3×10^−6^;1.2×10^−5^) for this donor (Table 1 and Figure 2).

**Table 1:** Frequency of deleted and duplicated alleles in sperm from three control donors.

	Deletion				Duplication			
	donor A	donor B	donor C	Pooled	donor A	donor B	donor C	Pooled
**Positive wells**	26	25	23	74	23	20	22	65
**Nb of recombinants**	30	29	26	85	26	22	25	74
**Total nb of alleles**		1.6E+6		4.8E+6		3.2E+6		9.6E+6
**λ**	1.9E−5	1.8E−5	1.6E−5	1.8E−5	8.1E−6	6.9E−6	7.8E−6	7.7E−6
**95% CI inf**	1.3E−5	1.2E−5	1.1E−5	1.4E−5	5.3E−6	4.3E−6	5.1E−6	6.1E−6
**95% CI sup**	2.7E−5	2.6E−5	2.4E−5	2.2E−5	1.2E−5	1.0E−5	1.2E−5	9.7E−6

When pooling the results from the three sperm donors, more robust estimates are obtained: the *de novo DPY19L2* deletion rate is estimated at 1.8×10^−5^ (95% CI: 1.4×10^−5^; 2.2×10^−5^), while the *de novo* duplication rate is estimated at 7.7×10^−6^ (95% CI: 6.1×10^−6^; 9.7×10^−6^) (Table 1). There is a significant approximately two-fold enrichment of deletions over duplications at the *DPY19L2* NAHR hotspot.

We investigated whether differential amplification efficiency between the deletion and duplication assays could explain the observed difference between deletion and duplication *de novo* rates. To this end, we performed a control experiment as described (see Methods). No significant difference in amplification efficiency was observed: the deletion-specific control PCR amplified 37 wells, and the duplication-specific PCR amplified 40 wells.

### Precise localisation of the recombined allele's breakpoints

Amplification of the LCRs in the deleted alleles had not been achieved in our previous study and the breakpoint minimal region had only been narrowed down to a 15 Kb region within the LCRs (8). Here we designed and validated PCR primers that amplify a 2 Kb product in deleted individuals only (Figure 1B). We quickly realised that mapping the breakpoints was complicated by the fact that many of the nucleotides that differed between LCR1 and LCR2 in the reference sequence were in fact not specific to one or the other LCR. Since mapping the breakpoints requires markers specific to each LCR, we decided to amplify and sequence the 2 Kb breakpoint region for each LCR in 20 control individuals. To achieve the specific amplification of LCR 1 and 2 we had to rely on the reference human genome sequence to design the primers. We had no way of confirming that the targeted LCRs were specifically amplified in control individuals, but no amplification was obtained when assaying twenty homozygous deleted patients, vouching for the specificity of the primers. We then amplified and sequenced LCR1 and 2 from a total of 20 control individuals: 10 of North African origin and 10 of European origin. Thirty-four nucleotides were indicated as specific to either LCR 1 or 2 in hg19 reference sequence but 14 of these were in fact arbitrarily found in the two LCRs (Table S4): we consider that these are non-LCR-specific single nucleotide polymorphisms (SNPs). The remaining 20 nucleotides were indeed LCR-specific: these 20 fixed markers were used to map the recombination breakpoints, and we used the 14 SNPs to establish a haplotype map of the patients' deleted alleles (Table S4).

**Figure 3:**
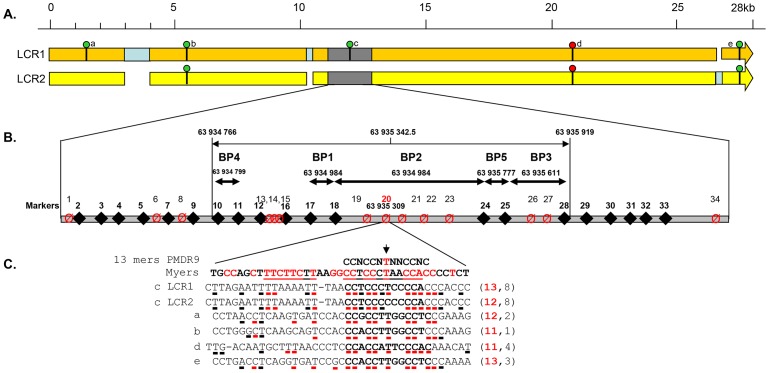
Details of the *DPY19L2* LCR1 and 2 and of the NAHR hotspot. (A) Detailed scaled representation of the 28.2 Kb LCR 1 (orange) and 27 Kb LCR2 (yellow). Pale blue rectangles correspond to sequences specific to one of the LCRs facing a gap in the other LCR. The presence of a 13 bp consensus PRDM9 recognition site (CCNCCNTNNCCNC) on LCR1 or LCR2 is indicated by a green circle when identified on the forward DNA strand and by a red circle when identified on the reverse strand (GTGGNNAGGGTGG). The LCR arrows point toward the chromosome 12 telomere. (B) The analysed recombination region is represented in grey. The positions of LCR-specific markers (diamonds and bold numbering) and variable nucleotides (crossed circles) are represented. Details of the markers' sequences and localisations are indicated in Table S2. The five identified breakpoints (BP1–BP5) are shown as double arrows. One PRDM9 consensus sequence is localised in the centre of BP2, the central and most frequent breakpoint. (C) The central nucleotide from the consensus sequence corresponds to one of the identified SNPs (snp 20). A perfect match for the consensus sequence is present on LCR1, while the central thimine is replaced by a cytidine in LCR2. The 39 nt surrounding the 5 matches to the PRDM9 consensus sequence identified in LCR1 and 2 (sites a–e) are compared with the consensus sequence described in Myers et al [8], [11]. Highly conserved nucleotides are red. For each locus the number of nucleotides identical to the consensus sequence is indicated on the right.

Allele-specific amplification of the deleted LCR was carried out on 15 homozygously deleted globozoospermia patients. Each amplification yielded a single 2088 bp product, while the PCR was negative for all the healthy controls tested (n = 20). We sequenced all the amplicons in order to better characterize the breakpoint region. Fourteen out of the 15 patients analysed were homozygous for all markers tested. Three different breakpoints (BPs) were identified based on the presence of the 20 invariant markers. The three recombination events (BP1–3) were included in a 1153 bp maximal region (Table S4 and Figure 3). The breakpoints could not be mapped more accurately for lack of nucleotides specific to each LCR. One patient was heterozygous for markers 13 and 14, indicating that this patient was heterozygous and carried two different deleted alleles (BPs 2 and 3). If we consider that the other 14 patients carried two recombined deleted alleles each, we have a total of 14 alleles with BP1 (between markers 17 and 18), 13 alleles with BP2 (between markers 18 and 24) and 3 with BP3 (between markers 25 and 28) (Figure 3B). The 14 identified SNPs were then used to map the different haplotypes in patients presenting the same breakpoint (Table S4). This shows the presence of a total of 7 distinct haplotypes, indicating that at least 7 recombination events are at the origin of our patients' pathology (15 patients). We also observe that 5 patients with BP2 have the same haplotype and that two groups of 3 patients with BP1 have the same haplotype, suggesting the presence of several founding deletions in our patients' population. This is not surprising as all our patients came from the same region (Tunis area) and a majority had related parents (often first cousins).

**Figure 4:**
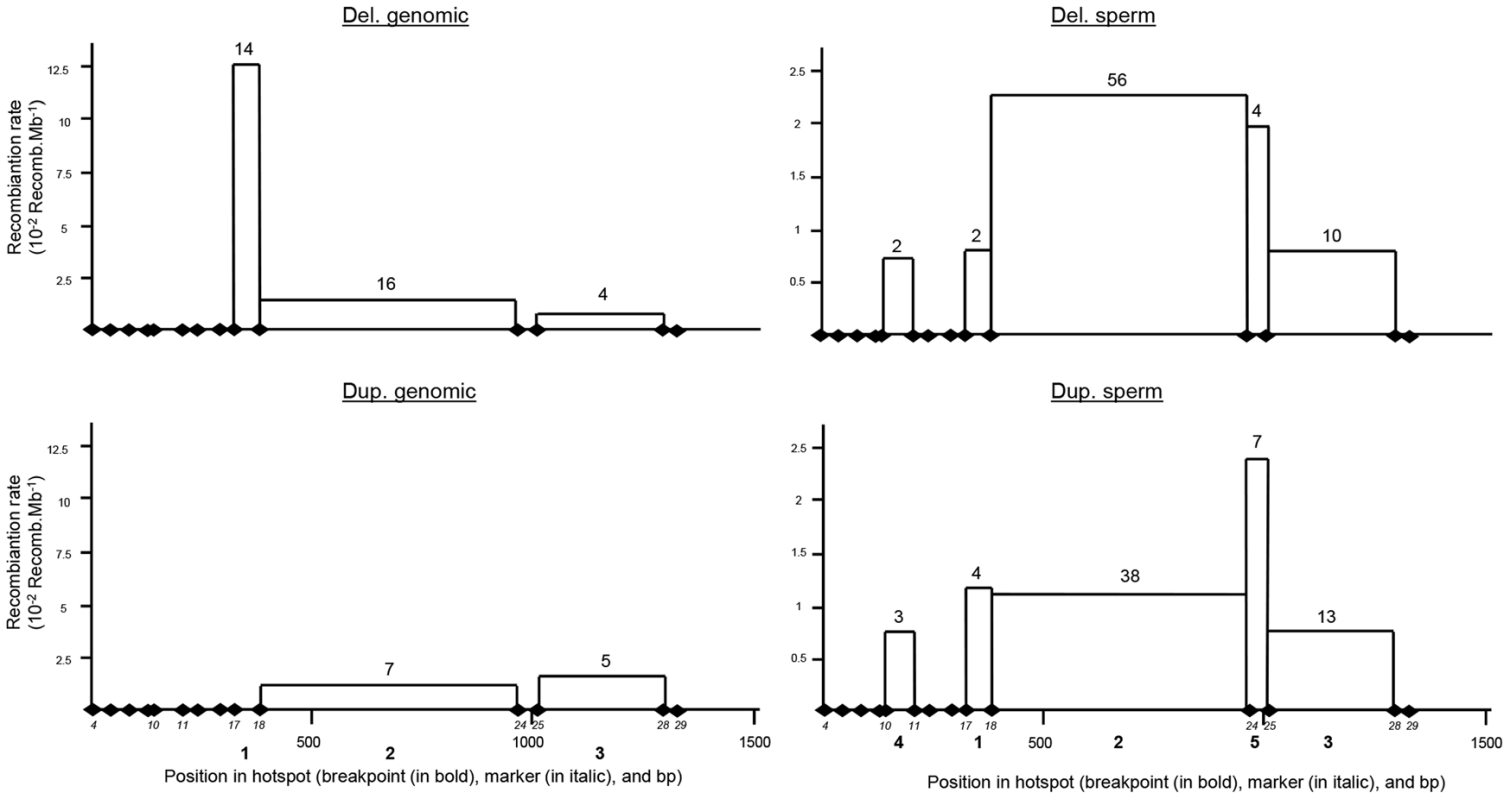
Distribution of deleted and duplicated breakpoints observed from somatic DNA (left two panels) and sperm DNA (right two panels). Somatic deletions were identified from sequence analysis of 15 homozygous deleted patients and two heterozygous deleted control individuals. Somatic duplications were identified from 12 positive control individuals. Data from sperm were pooled from three control donors.

One and three deletions were identified respectively in the 300 individuals analysed by PCR and in the 1699 Grenoble-Lyon array CGH patients group. There were 3 occurrences of BP2 and 1 of BP3. Overall, including the globozoospermia patients, a total of 34 somatic deleted alleles were examined, resulting in the detection of three different recombination breakpoints. Fourteen alleles (41.2%) had a deletion between markers 17 and 18 (BP1), 16 alleles (47.0%) between markers 18 and 24 (BP2), and 4 alleles (11.8%) were recombined between markers 25 and 28 (BP3) (Figure 4 top left).

Two and fifteen genomic duplicated alleles were detected respectively in the 300 control individuals analysed by PCR and in the Grenoble-Lyon array CGH patients. Only 12 duplicated alleles could be sequenced (for lack of DNA from 3 control subjects and because two of the subjects had breakpoints falling outside the range of the duplication-specific PCR). Seven alleles (58.3%) corresponded to the reciprocal alleles of deletion 2 (BP2) with a recombination between markers 18 and 24, and 5 alleles (41.70%) corresponded to the reciprocal alleles of deletion 3 (BP3) with a recombination between markers 25 and 28 (Figure 3B).

The position of the meiotic recombination events (deletion and duplication) obtained from three sperm donors were also characterized by DNA sequencing. A total of 74 *de novo* deleted alleles and 65 *de novo* duplicated alleles were sequenced. All recombination events (from both duplications and deletions) clustered into five breakpoints (Figure 4). Two of them are new (BP4 and BP5) i.e. not previously identified in globozoospermic patients or in the CGH control cohort. The number and percentages of deleted and duplicated breakpoints respectively are: BP1: 2 (2.7%) and 4 (6.1%); BP2: 56 (75.7%) and 38 (58.5%); BP3: 10 (13.5%) and 13 (20%); BP4: 2 (2.7%) and 3 (4.6%) and BP5: 4 (5.4%) and 7 (10.8%) (Figure 4). BP2 is by far the most frequent BP, followed by BP3, explained by the fact that these two breakpoints correspond to the largest regions. Interestingly in sperm, the distributions of the deleted and duplicated breakpoints are quite similar. This is logical as the duplicated alleles are expected to be the reciprocal alleles of some of the deleted alleles. In genomic DNA the correlation is not as good, and we note that the frequency of the deleted BP1 is particularly high. Most of the deleted alleles come from globozoospermia patients (and a few detected in CGHarray patients) most of whom were recruited in Tunis. As suggested by the shared haplotypes observed between some deleted patients (Table S4) a founder's effect is likely to account for some of the most frequent deletions, in particular BP1.

### PRDM9 genotyping of the sperm donors

Sequencing of the PRDM9 ZF array was performed in the 3 sperm donors. All three donors were homozygous for the A allele which represents over 90% of the European alleles. It comprises 13 copies of the 84-bp ZF repeat that binds the 13-bp Myers recombination motif [12], [14]. This result is concordant with the ethnicity of the donors.

### Detailed analysis of LCR1 and 2

A comparison of the two LCRs is presented in Figure 3A. The illustration was produced from the results of a megablast search (http://blast.ncbi.nlm.nih.gov/Blast.cgi?PROGRAM). All identified recombined alleles (n = 185) cluster between markers 10 and 28 within a 1153 bp region. This recombination hotspot is roughly located in the middle of the 28 Kb LCR (Figure 3A). Five 13 bp PRDM9 consensus recognition sites (CCNCCNTNNCCNC) are present along the LCR (Figure 3A). One of these sites is located in the centre of the 1153 bp hotspot (less than 35 nt away from the hotspot median position) (Figure 3B). We note that the most central BP (BP2) which encompasses the 13 bp site, represents 117 out of 185 recombined alleles or 63% of the detected recombined alleles (Figure 4). Given that five PRDM9 consensus recognition sites are found within the 28 Kb LCR1 sequence, the probability that a site would occur by chance less than 35 bp away from the centre of the hotspot is 1−(1−5/28000)^70^ = 0.012.

The Thymin at the centre of the consensus recognition site (CCNCCNTNNCCNC) was present only in the reference sequence of LCR1. Sequence analysis of our control individuals showed that this nucleotide was in fact a SNP (Marker 20 in Figure 3 and Table S4) with a T allele frequently found in both LCR1 and LCR2 (Table S4). In our globozoospermia patients we observed that all patients with the BP1 (with marker 20 located after the breakpoint thus on LCR2 sequence) have the T allele, indicating the presence of a T allele on LCR2 of the original unrecombined allele (Table S4). Conversely patients with BP3 (with marker 20 located before the breakpoint thus on LCR1 sequence) have the C allele indicating the presence of a C allele on LCR1 of the original unrecombined allele. All patients with BP2 have the C allele. As marker 20 is located within the breakpoint maximal sequence we can only conclude that at least a C allele was present on either LCR1 or LCR2 of the original unrecombined allele.

We sequenced LCR1 and LCR2 of our three sperm donors and realised that all where homozygous for the C allele at both LCR1 and LCR2, suggesting that the presence of the thymine in the CCNCCNTNNCCNC consensus sequence is not necessary to initiate recombination in the *DPY19L2* LCR central region. Myers et al. [8], [11] indicated that although the core 13-mer recognition sequence was associated with recombination hotspots, the recognition motif extended beyond the core sequence with preferentially associated nucleotides identified within a 39 bp sequence encompassing the PRDM9 core sequence. We therefore aligned this extended motif with the sequence of the 5 PRDM9 motifs identified within the LCR (Figure 3C). We observe a good correlation within all 5 sequences, especially for the nucleotides that had been shown to be significantly associated with hotspots (indicated in red in Figure 3C). We also observe that the sequence central to our recombination hotspot (motif c) presents the highest homology (53%) with Myers' extended recognition sequence (Figure 3C).

## Discussion

It appears paradoxical that *de novo* deletions are produced twice more frequently than *de novo* duplications during meiosis, while duplicated alleles are three times more frequent than deleted alleles in the general population. We investigated whether this could be explained parsimoniously through the combined effects of selection and mutation. Men carrying a homozygous deletion of *DPY19L2* are 100% infertile, but currently there is no evidence that a heterozygous deletion of *DPY19L2* causes a phenotype or that homozygous women are affected. Additionally, the deleted allele is rare. Under these assumptions, according to the General Selection Model (GSM), natural selection results in a decrease in the frequency of the deleted allele of approximately q^2^/2 per generation, where q is the frequency of the deleted allele (see Methods). Given that the deleted allele has a frequency of 1.7×10^−3^ (95% CI: 1.2×10^−3^; 2.5×10^−3^) in the general population according to our combined control data, the GSM predicts that this frequency decreases by 1.5×10^−6^ (95% CI: 7×10^−7^;3.1×10^−6^) per generation. Conversely, deleted alleles are produced *de novo* by NAHR at an estimated rate of 1.8×10^−5^ (95% CI: 1.4×10^−5^; 2.2×10^−5^) according to our digital PCR data. Assuming the allele frequency is at an equilibrium, these two rates should balance out. In fact they are somewhat similar but the 95% confidence intervals do not overlap. However the CIs only represent the uncertainty induced by the sampling procedure, i.e. the fact that the allele frequency and recombination rate are estimated from a sample of the whole population: they do not take into account experimental biases or imperfections that may exist at various steps. In addition, the GSM is a theoretical model that assumes an infinite population size and panmixia, whereas in practice stochastic effects and population structure (including for example any potential consanguinity or local founder effects) come into play. These could result in a significantly increased impact of purifying selection on the deleted allele, so that the frequency decrease resulting from selection and the *de novo* production of deleted alleles through NAHR may in fact cancel out.

Alternatively, it is possible that heterozygously deleted men suffer a fitness penalty. This can be taken into account within the GSM, and one can calculate the relative fitness of heterozygous individuals such that the GSM-predicted decrease of the deleted allele's frequency compensates the measured NAHR-induced production of new deleted alleles. In fact, assuming women are not affected, a 98% relative fitness of heterozygous men is sufficient (see Methods). Such a small effect could have easily remained undetected, and this scenario cannot be ruled out. This potential selection could be caused by meiotic segregation distortion as was observed for the T/t mouse locus [34]. Finally we only studied the recombination rate in male germ cells and we cannot exclude the possibility that the frequency and ratio of deletion and duplication might be different in female gametes.

All in all we believe the rates are reconcilable: whether the discrepancy observed when assuming heterozygous individuals have no phenotype is due to imperfections in the data and/or to population structure which disrupts the theoretical GSM model, or whether heterozygously deleted men suffer a small fitness penalty, we propose that the frequency decrease due to purifying selection and the *de novo* production of deleted alleles through NAHR cancel out, and that the frequency of the deleted DPY19L2 allele is today at a selection-recombination equilibrium in the population. On the other hand, to the best of our knowledge there is no evidence that the duplicated *DPY19L2* allele is either deleterious or advantageous. We therefore assume that the duplicated DPY19L2 allele is not under selection, so its frequency can increase in the population by recurrent NAHR. This resolves the paradox.

Liu and colleagues (2011) proposed that the frequency of NAHR occurring between two paralogous LCRs was proportional to the LCR length and sequence homology but inversely proportional to the distance between the LCRs [6]. The authors logically proposed that the probability of ectopic chromosome synapsis increases with LCR length, and that ectopic synapsis is a necessary precursor to ectopic crossing-over. Here we measured that the average rate of *de novo* recombination (deletion plus duplication) by NAHR at the *DPY19L2* recombination hotspot was 2.6×10^−5^. This rate is higher than what was measured at other loci such as the Williams-Beurren syndrome (WBS) locus or the LCR17p locus [4]. In our case the relatively small LCR size (28 Kb) is compensated by the proximity of the repeats (200 Kb) compared with much greater distances separating the paralogous LCRs for WBS and LCR17p. *DPY19L2* LCR1 and 2 also present a very high sequence identity (98%) which could also reinforce their synapsis and recombination. Our results are in agreement with previous work suggesting that the distance separating the two LCRs, as well as their sequence homology and length are parameters likely influencing recombination frequency.

We observed that >90% of *DPY19L2* NAHR events occurred within a 1.2 Kb region located in the centre of the 28 Kb LCR, suggesting the presence of a pro-recombination sequence within this hotspot. Myers et al. (2008) have characterized a degenerate 13 bp sequence motif that is present in approximately 40% of the identified human hotspots and which constitutes a *PRDM9* recognition signal [12]–[14]. *PRDM9* codes for a zinc finger array which catalyses the trimethylation of the lysine 4 of histone H3 (H3K4me3) [11]. This PRDM9-mediated post-translational histone modification likely initiates the recruitment of the recombination initiation complex, creating a favourable chromatin environment and allowing access of SPO11 to the DNA. SPO11 then initiates the formation of double-strand breaks (DSBs) which will be repaired by homologous recombination [35]. Here we identified a hotspot of NAHR located in the centre of a 28 Kb LCR. We showed that a PRDM9 13-mer recognition sequence is present at the epicentre of all the identified breakpoints. We however realised that the thymine, central to the 13-mer motif (CCNCCNTNNCCNC), was a T/C SNP, each nucleotide being found arbitrarily within LCR1 or LCR2. Following this observation one can wonder if recombination events at the DPY19L2 hotspot occur preferentially in the presence of fully matching PRDM9 13-mer alleles. We measured the frequency of *de novo* recombination in sperm from three donors. As it happens, sequencing revealed that all three were homozygous for the C allele on both LCR1 and LCR2. This indicates that, at this locus, the presence of the 13-mer exact match is not necessary to initiate recombination. This observation is concordant with what was described previously at different loci and confirms that PRDM9 tropism for the 13-mer recognition site might not be very strong and/or that other mechanisms also intervene in the choosing of double strand break localization [12], [15]. One explanation can come from the extended sequence surrounding the 13-mer motif. Myers and colleagues (2008) [10] described a 39 bp pro-recombination sequence encompassing the 13-mer motif. We observe a greater than 50% sequence identity for the complete 39-mer sequence, indicating that a good match to the extended motif might be at least as important as a perfect match of the core 13-mer motif.

We identified a total of 5 distinct breakpoints (BP), all localized within a 1.2 Kb region located in the centre of the 28 Kb LCR. Others have described the localization of the deletions of globozoospermia patients [22]. They described a total of 9 separate BPs in the *DPY19L2* LCR. Looking at the precise localizations of the described BPs, we noticed that the nucleotides used to delimit BPs 1–6 in that study are in fact nucleotides that we identified as SNPs (markers 19–23 and 26), which strongly questions the validity of the BP localization in that study. Reanalyzing the presented data and using LCR-specific markers only, we conclude that Elinati et al. (2012) BPs 1, 2, 4, 5, 6 fall within the boundaries of “our” BP2 and that “their” BP3 corresponds to “our” BP3. This illustrates the difficulty in precisely identifying the localization of BPs and demonstrates that this can only be achieved with a high level of confidence after confirmation that the markers used to define the BP positions are indeed locus-specific. From our reanalysis, Elinati et al. (2012) identified deletions in 27 globozoospermia patients, 23 had our BP2, one had BP3 and one had a BP that fell just outside of our studied region. These results thus confirm the importance of the recombination hotspot described here. Two additional BPs (BP8 and 9) were also identified in Elinati's study which fell well outside of our recombination hotspot. This might constitute a second, less frequent recombination hotspot within the LCRs. We noticed that these two BPs are located 1200 bp telomeric from the 13-mer PRDM9 site d (as indicated in Figure 3A). Thus this second putative hotspot is further away from a consensus 13-mer motif than our hotspot (the greatest distance of the BPs we identified from the 13-mer is 600 bp), but we can question again the accuracy of the positioning of these two breakpoints. Here, while analyzing the array CGH recombined patients we identified two recombined alleles which did not fall within our studied BP area. It is possible that these recombination events are also located within this second putative hotspot.

With the *DPY19L2* locus we believe that we have a good model to study the effect of the PRDM9 recognition site on NAHR. We plan to accurately position the yet uncharacterized BPs in relation to other PRDM9 sites. We are also currently screening an anonymized sperm bank to identify donors that are homozygous for the central 13-mer PRDM9 recognition T allele and/or who present rarer PRDM9 alleles to investigate how the recombination rate is affected by both the PRDM9 genotype and the extended PRDM9 recognition motif. We believe that although much work remains to be done, our study illustrates and consolidates the hotspot models described previously. In a moving environment we can imagine that the central region of the LCR will have the most opportunities to synapse with its paralogous sequence. The presence of an extended PRDM9 recognition motif in the centre of the LCR then very likely contributes to DSB and NAHR. The combination of these parameters therefore probably explains why approximately 90% of the breakpoints occurred within a few hundred nucleotides from the most centrally located PRDM9 recognition site.

## Materials and Methods

### Ethics statement

All patients, family members and anonymous DNA and sperm donors gave their written informed consent, and all national laws and regulations were respected. Ethical approval was obtained from Grenoble CHU review board.

### Information on patients and control individuals

We previously reported that 15 out of 20 patients with globozoospermia had a homozygous deletion of the DPY19L2 region [17]. These patients are included in this study. All patients are unrelated apart from two who are brothers. All patients originated from North Africa (Tunisia, n = 12; Morocco, n = 2 and Algeria, n = 1).

Array CGH data from a total of 1699 control anonymous individuals were re-analysed. These analyses had been carried out as a diagnosis for syndromic mental retardation either at Grenoble or Lyon's hospital. As our aim was to identify *DPY19L2* centred CNVs in this cohort of patients and since there is no known link between *DPY19L2* and mental retardation, we believe that this cohort can serve as a control in this study. All individuals agreed to the anonymous use of their DNA in genetic studies and signed an informed consent. The fertility and ethnic origin of these individuals was not documented. All were French citizens. We estimate that in excess of 90% of these individuals are of European origin and that the vast majority of the others are of North African origin.

There was no gender selection but this cohort contained approximately 2/3^rd^ of males.

Array CGH results from these patients were scrutinized for the *DPY19L2* region.

Three hundred control individuals were analysed independently with recombinant *DPY19L2*-specific PCR (deleted and duplicated) to identify deleted and duplicated alleles. One hundred and fifty individuals originated from North Africa (Algeria, Morocco, and Tunisia) and 150 originated from Europe. All individuals gave their informed consent to constitute an anonymous DNA bank. Non-recombined LCR1 and 2 of twenty of these individuals were amplified and sequenced to identify LCR-specific SNPs. There was no gender selection and this cohort contained a similar number of males and females.

Lastly the *DPY19L2* CNV was also analysed from array CGH data available from web servers [29]–[33] for a total of 6575 control individuals, mainly from the Database of Genomic Variants (http://projects.tcag.ca/variation/). Most of these individuals originated from Europe (75%), Africa (18%) or Asia. Individual CNV could however not be linked to a particular individuals and its geographical origin. The location of the breakpoints of each CNV located in the *DPY19L2* locus was scrutinised to establish if they were located within the LCR and hence were caused by NAHR (Tables S2 and S3).

### DNA extraction

Genomic DNA was extracted either from peripheral blood leucocytes using a guanidium chloride extraction procedure [36] or from saliva using Oragene DNA Self-Collection Kit (DNAgenotech, Ottawa, Canada).

Sperm DNA was extracted from 2 ml of semen which were transferred to a 25 ml Falcon Tube (BD Biosciences). Ten ml of PBS was added, mixed gently and centrifuged at 3,000 rpm for 5 minutes. Supernatant was discarded and the pellet was resuspended again in 10 ml of PBS, mixed and centrifuged as before. Pellets were then resuspended in 1 ml digestion buffer (NTE buffer 0.5 mM NaCl, 10 mM Tris-HCl pH 7.5, 5 mM EDTA, pH 8 (100∶10∶1), 0.4% SDS), 25 µl of 10 mg/ml proteinase K solution (Sigma) were added and the mix was incubated overnight at 42°C with occasional mixing. Three hundred microliters of the contents of each Falcon tube were transferred into SafeLock tubes (Eppendorf). An equal volume of phenol/chloroform/isoamyl alcohol (25∶24∶1) was added and mixed gently until emulsified. The tube was centrifuged at 3,000 rpm for 5 minutes. We repeated this process a second time, adding an equal volume of chloroform/isoamyl alcohol. The upper aqueous layer was transferred into a clean Eppendorf tube. The aqueous layers from the two phenol/chloroform extractions were combined and an ethanol precipitation was performed: 25 µl 3M sodium acetate pH 5.4 and 1 ml 100% ethanol were added to the aqueous phase, mixed gently and centrifuged as before. The pellets were washed twice with 70% ethanol and finally resuspended in 300 µl TE buffer (10 mM Tris-Cl pH 8.0, 0.1 mM EDTA, pH 8.0 (10∶1)) by incubating overnight at 50°C with gentle shaking.

### Information about the sperm donors

DNA was extracted from three fertile anonymous donors of European origin with normal sperm parameters and of similar age (between 30 and 35 years old). In each case a spermogram was realised according to WHO's 2010 guidelines [37]. Sperm concentration ranged between 60–120×10^6^ spz/ml, with, in each case less than 1×10^6^ leucocytes/ml. We therefore considered that the presence of this small percentage of leucocytes had a negligible effect on the quantification of the sperm (only) DNA and on the ensuing calculations.

A molecular analysis was carried out to determine PRDM9 ZF array genotype. PCR amplification and sequencing of the PRDM9 ZF array were performed using primers and protocols as described previously ([12]. PCR and sequencing primer sequences are listed in the Table S1.

### Amplification and sequencing of the LCRs

All primers were designed to have at least their 3′ nucleotide specific to the LCR of interest (Table S1). PCR primers were designed to amplify specifically LCR 1 or LCR 2, in order to perform a sequence comparison of the two LCRs. For each recombined LCR locus (resulting from deletion or duplication), two sets of specific primers were designed (Figure 1B). The external primers (long primers) were used for sequencing analysis. They were also used as an outer primer for the digital PCR that was devised to measure the rate of *de novo* recombination in sperm. The short internal primers (SI) were used in duplex with *RYR2* primers that were used as a positive amplification control. These two sets of primers were used to detect the presence of recombined alleles in the 300 control individuals. They were also used as inner primers for the digital PCR.

PCR amplification was carried out on an Applied Biosystems genAmp 2700 thermocycler. Due to the high sequence homology between the two LCRs, the use of a precise annealing temperature was critical. The same thermocycler had to be used throughout the study as small variations in block temperature could introduce discrepancies in the amplification. Both the long and short PCR cycles were preceded by a 7 minutes denaturation at 95°C and followed by a 10 minutes elongation at 72°C. The specific annealing temperature of each primer set is indicated in Table S1. Thirty-five cycles were carried out for the long PCRs, with 30 seconds of denaturation at 95°C, 30 seconds of annealing and 2 minutes of elongation at 72°C. Forty-five cycles were carried out for the short PCRs with 30 seconds of denaturation at 95°C, 20 seconds of annealing and 2 minutes of elongation at 72°C.

We performed the long and short PCRs in 1× Takara Ex Taq buffer (Takara), 250 µM dNTPs (Takara dNTP mixture), 300 nM each primer, 1 unit Takara Ex Taq Takara) with 200 ng of somatic DNA in a total volume of 25 µl.

All sequences (native LCR 1 and 2 and deleted and duplicated LCRs) were carried out with BigDye Terminator v3.1 (Applied Biosystems Courtaboeuf, France) on an ABI 3130XL (Applied Biosystems, Courtaboeuf, France).

Oligonucleotide array CGH was performed with the Agilent 105K or 180K Human Genome CGH Microarray (Agilent Technologies, Santa Clara, CA, USA) (Hospices Civils de Lyon array CGH Platform and CHU Grenoble array CGH Platform). Extracted DNAs were labelled according to the instructions of the supplier and incubated overnight. The samples were purified and hybridised as described previously [17].

Graphical display and analysis of the data were performed with the Agilent DNA Analytics software version 4.0.81 (statistical algorithm: ADM-2, sensitivity threshold: 2.5, window: 0.5). A value of zero represents equal fluorescence intensities between sample and reference DNA. Copy-number losses shift the value to the left (≤−1), and copy-number gains shift it to the right (≥0.58).

The design of the MLPA probes, MLPA reaction and data analysis were performed according to the recommendation of the MRC-Holland synthetic protocol (www.mlpa.com) and as described in Coutton et al. (2012) [25].

### Sperm assay design and digital PCR for sperm NAHR breakpoints mapping

We designed two nested LCR-specific PCRs as described in the PCR section. In addition, we designed a TaqMan dual labeled probe (Table S1) to allow the second step of the nested PCRs to be run on Biorad iCycler IQ real time PCR detection. We tested each recombinant-specific combination of primers for specificity and sensitivity on negative and positive (*DPY19L2* deleted and duplicated) control blood DNA (Figure 1B). Each of the two rearrangements was assayed on DNA extracted from three unrelated sperm donors. Each donor was confirmed to carry two copies of *DPY19L2* by MLPA analysis (data not shown). We note that our assay will not distinguish triplications of the DPY19L2 locus, which are likely to occur at extremely low frequencies.

We performed the first LCR-specific PCR (long PCR) in 1× Takara Ex Taq buffer (Takara), 250 µM dNTPs (Takara dNTP mixture), 300 nM of each primer, 1 unit Takara Ex Taq (Takara), using sufficient copies of template DNA to give approximately 24 positive wells per 96-well plate (exact quantities determined empirically by successive dilutions) and 2.5 mM MgCl2, in a total volume of 50 µl. Following thermal cycling we incubated 10 µl of the long PCR products with 5 µl of Exosap-IT PCR Clean-up Kit (GE Healthcare) for 15 min at 37°C to digest the long PCR primers followed by enzyme inactivation at 80°C for a further 15 min. Two µl of 10× diluted long PCR products was used as a template in the second PCR (short PCR). In the short PCR we used the same concentrations of buffer, dNTPs, primers and enzyme as in the Long PCR, but the total volume was 25 µl and we added a dual-labeled probe (final concentration 250 nM; Eurofins MWG Operon) (Table S1). To map the locations of breakpoints we re-amplified wells that we had previously identified as positive in the long PCR plate using the short primers and sequenced these amplicons.

The quantity of input sperm DNA was experimentally determined by serial dilutions to obtain approximately 24 positive breakpoint-specific amplifications per 96-well plate. The number of positive amplifications was then counted to estimate the number of recombinants in the input sperm. Each well contains a sample drawn from the input DNA without replacement, hence the number of recombinants in a given well is appropriately modeled using a hypergeometric distribution. We note that this hypergeometric distribution has often been approximated in the literature by Poisson (6, 22) or binomial (23) distributions, but although such approximations are acceptable we find no need for them in this study, as the direct calculation is simple. Indeed, using the hypergeometric distribution the probability that a well contains no recombinants is:
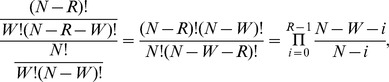
where N is the total number of copies of chromosome 12 in the input DNA (i.e. 1.6×10^6^ for the deletion assay and 3.2×10^6^ for the duplication assay, see Results section on digital PCR), W = N/96 is the number of copies per well, and R is the total number of recombinants. The value of R such that this probability is closest to the observed ratio of negative wells (i.e. one minus the fraction of wells that produced a positive amplification) is easily found by tabulation. This leads to an estimation of the *de novo* recombination rate λ = R/N, and a 95% confidence interval is calculated by modeling the initial dilution to obtain the input DNA using the binomial distribution (with binom.test in the R stats package, http://www.r-project.org).

In order to evaluate the amplification efficiency of our duplication/deletion assays, we used as positive controls genomic DNA from one heterozygous duplicated individual and from one heterozygous deleted individuals. We believe that this type of control is more accurate than the use of cloned recombinant deleted and duplicated alleles as this reduces dilution factors. More importantly it reproduces faithfully the possible inhibitions due to the presence of the over majoritarian non-target genomic DNA or the potential amplification of homologous sequences that are present in the actual quantifying experiments.

The DNA concentration was measured by Nanodrop (ThermoScientific) and DNA quality was evaluated using an agarose gel electrophoresis (0.8%). No smear or fragments were observed. Considering that a human diploid genome represents 6 pg of DNA, we performed serial dilutions of the duplication and deletion controls to obtain a concentration of 1.5 pg/µl. One microliter of each solution was aliquoted in a 96-well plate, so that approximately 25% of the wells are expected to contain a recombinant allele (as we used heterozygous controls who carry only one copy of the deleted or duplicated alleles). The number of positive wells was then counted when amplifying deleted and duplicated DNA.

### Calculations with the General Selection Model

Given a locus with two alleles (e.g. wild-type DPY19L2 allele and deleted allele), and noting q the frequency of the minor (deleted) allele, the GSM predicts the change in allele frequency Δq at each generation given the relative fitness of each genotype. In our case the homozygous wild-type is used as a reference (fitness 1), and the homozygous deleted men are known to be 100% infertile while the deletion is considered to have no effect in women, hence the fitness of the homozygous deleted genotype is 0.5. Let W be the relative fitness of the heterozygous genotype, and p = 1-q the frequency of the wild-type allele. Note that q≈1.7×10^−3^, hence p≈998×10^−3^. The GSM therefore simplifies to: *Δq≈−q[W(q−p)+p−q/2]*.

In the first scenario, heterozygous individuals are assumed to have no phenotype, hence W = 1 and the equation simplifies to: 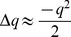.

In the second scenario, we no longer assume W = 1 and instead wish to calculate the value of W such that the GSM-predicted Δq exactly compensates the *de novo* rate of production of deleted alleles through NAHR, i.e. Δq = −1.8×10^−5^. Turning the previous equation around, we obtain: 
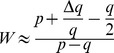
. Substituting the values of p, q and Δq, this yields W = 0.99. Assuming that heterozygous women have no phenotype, we finally obtain a relative fitness of 98% for heterozygous males.

## Supporting Information

Figure S1 Identification of a duplication of the *DPY19L2* locus by array CGH. Array-CGH analyses showed a 130 kb gain extending from base 63,947,732 to 64,078,229 in chromosome 12q14.2. Coordinates of variations or probes (y-axis) are based on the UCSC GRCh37/hg19 assembly.Graphical overview and analysis of the data were obtained with the Genomic Workbench software, standard edition 6.5 (Agilent) with the following parameters: aberration algorithm ADM-2, threshold 6.0, fuzzy zero, centralisation and moving average window 0.5 Mb.The value of zero (x-axis) represents equal fluorescence intensity ratio between sample and reference DNA. Copy-number gains shift the ratio to the right (positive values). Three adjacent probes located at the *DPY19L2* locus are duplicated in the analyzed patient and the mean log2 ratio was +0.53 according to the Alexa 5 deviation with a mirror image.
(GIF)

Table S1 Sequence of the PCR primers, position (Hg19), size of the amplified products (between brackets), hybridization temperature (Hyb.). The position of the primers is illustrated in Figure 1B.
(DOC)

Table S2 Sequence of the recombination hotspot region of control subjects (10 Europeans and 10 North Africans) for the identification of LCR-specific markers and determination of the precise localisation of the breakpoints of 15 globozoospermia patients.
(DOC)

Table S3 Number and percentage (%) of recombined alleles with breakpoints located within (inside) or outside of the LCR.
(DOC)

Table S4 Sequence of the recombination hotspot region of control subjects (10 Europeans and 10 North Africans) for the identification of LCR-specific markers and determination of the precise localisation of the breakpoints of 15 globozoospermia patients. The first column indicates the reference number of the identified variants as shown in Figure 2. Markers that are LCR-specific (i.e. homozygous and invariant within each LCR across all controls, but differing between LCR1 and LCR2) according to the results obtained from the 20 sequenced control individuals (columns 5–8) are indicated in larger bold lettering. Nucleotides that are not LCR-specific are considered as SNPs. The markers' sequence in each LCR according to the Hg19 reference sequence is indicated column 4. In patients, the presence of the Hg19 reference nucleotide is indicated by a cross. When a single nucleotide is detected, the patient is considered homozygous at that position. Because the rows are color-coded, with alternating grey and white rows corresponding to the Hg19 reference nucleotide for LCR1 or LCR2 respectively, and because bold crosses correspond to validated LCR-specific markers, the recombination breakpoints can be easily visualized. For each patient a vertical stretch of bold crosses in grey rows (displayed in orange rectangles) shows non-recombined genetic material coming from LCR1. This is followed by a stretch of bold crosses in white rows (displayed in yellow rectangles), which shows non-recombined DNA from LCR2. For each patient the breakpoint's localisation is inferred when his genotype shifts from LCR1- to LRC2-specific markers. Unboxed regions therefore correspond to breakpoint maximal regions. Patients 1–7 breakpoints are located between markers 17 and 18 (BP1). Patients 8–13 BPs are located between markers 18 and 24 (BP2). Patient 14 is the only heterozygous patient, with BP2 and a breakpoint between markers 25 and 28 (BP3). Patient 15 is homozygous for BP3. SNPs differing between patients with the same breakpoints are highlighted with a blue background. This indicates the presence of 3, 2 and 2 distinct haplotypes for BP1, BP2 and BP3 respectively. Overall this indicates the presence of 7 distinct haplotypes, so that the occurrence of at least 7 separate recombination events within our series of 15 patients can be inferred.
(XLS)
