# Supplementary figures and images for: Fine Characterisation of a Recombination Hotspot at the DPY19L2 Locus and Resolution of the Paradoxical Excess of Duplications over Deletions in the General Population
Source: PLoS Genet. 2013 Mar 21;9(3):e1003363. doi: 10.1371/journal.pgen.1003363 (PMC3605140; doi:10.1371/journal.pgen.1003363)

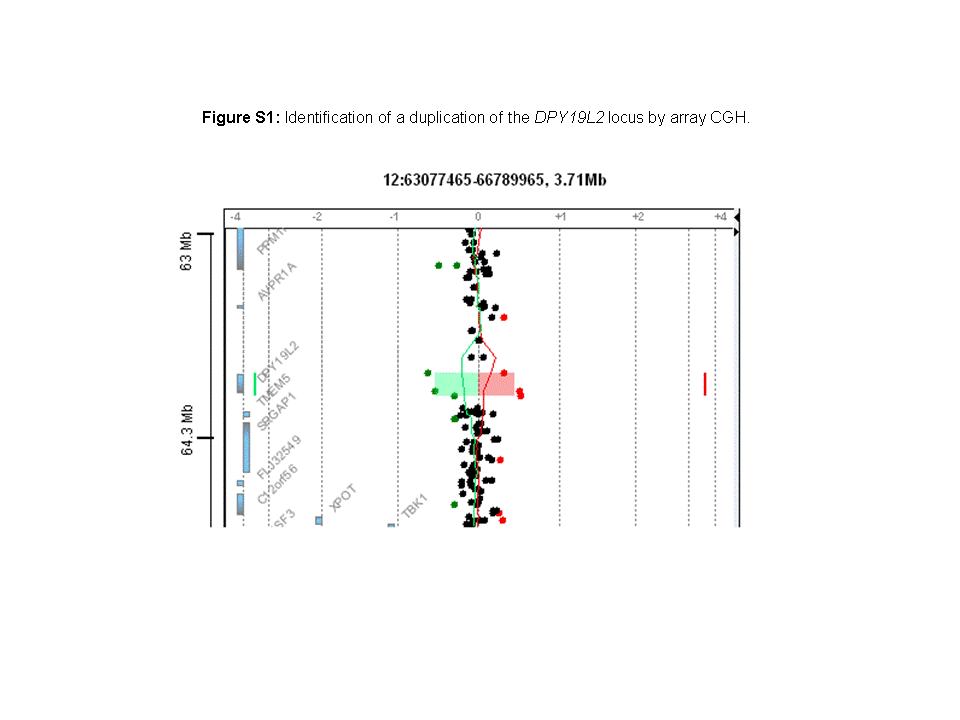

Supplement: Figure S1 — Identification of a duplication of the DPY19L2 locus by array CGH. Array-CGH analyses showed a 130 kb gain extending from base 63,947,732 to 64,078,229 in chromosome 12q14.2. Coordinates of variations or probes (y-axis) are based on the UCSC GRCh37/hg19 assembly.Graphical overview and analysis of the data were obtained with the Genomic Workbench software, standard edition 6.5 (Agilent) with the following parameters: aberration algorithm ADM-2, threshold 6.0, fuzzy zero, centralisation and moving average window 0.5 Mb.The value of zero (x-axis) represents equal fluorescence intensity ratio between sample and reference DNA. Copy-number gains shift the ratio to the right (positive values). Three adjacent probes located at the DPY19L2 locus are duplicated in the analyzed patient and the mean log2 ratio was +0.53 according to the Alexa 5 deviation with a mirror image. (GIF) [file pgen.1003363.s001.gif]
