# Supplementary material for: Fine Characterisation of a Recombination Hotspot at the DPY19L2 Locus and Resolution of the Paradoxical Excess of Duplications over Deletions in the General Population
Source: PLoS Genet. 2013 Mar 21;9(3):e1003363. doi: 10.1371/journal.pgen.1003363 (PMC3605140; doi:10.1371/journal.pgen.1003363)
Supplement: Table S1 — Sequence of the PCR primers, position (Hg19), size of the amplified products (between brackets), hybridization temperature (Hyb.). The position of the primers is illustrated in Figure 1B. (DOC) [file pgen.1003363.s002.doc]

Table S1 : details of the PCR primers.

| **Locus amplified** | **5’ to 3’ DNA sequence** | **Starting position (5’)and Size of amplicon (nt)** | **Hyb. (°C)** |
| --- | --- | --- | --- |
| 1) Long deletion specific  5’L1  3’L2 | TAGACTCTTCTGGGAAAGGTATTATCG  CCAAGGAAATCGAAGACGCT | chr12:63,934,316  chr12:64131430(2088) | 64 |
| 2) Long LCR1 specific  5’L1  3’L1 | TAGACTCTTCTGGGAAAGGTATTATCG CAAGGAAATCGAGGATGCC | chr12:63,934,316  chr12:63936360(2087) | 55 |
| 3) Long LCR2 specific  5’L2  3’L2 | ACTCTTCTGGGAAAGAAGAGAA  CCAAGGAAATCGAAGACGCT | chr12:64,129,361  chr12:64,131,430 (2089) | 55 |
| 4) Short Inner deletion specific  5’SI1  3’SI2 | ATAATCTGTAATTTCCACTGCATTCAG  GGCACAGCTGCCAGCATTC | chr12:63,934,614  chr12:64,131,009 (1392) | 60 |
| 5) Short duplication specific  5’SI2  3’SI1 | ATAATCTGTAATTTCTACTGCATTCTA  GGCACAGCTGCTAGCATTT | chr12:64,129,654  chr12:63,935,989 (1394) | 60 |
| 6) Long duplication specific  5’L2  3’L1 | TAGACTCTTCTGGGAAAGAAGAGAA  CCAAGGAAATCGAGGATGCCGA | chr12:64,129,358  chr12:63,936,381 (2085) | 64 |
| 7) Positive control primers  5RYRex40  3RYRex42 | TAGGCACAGAGTGAGAGGGTCAAG  GCAAATTAGTCTCCTTCTGGTTGG | chr19:38,986,748  chr19:38,987,714 (990) | 60 |
| 8) Dpy19l2 Taqman | 6FAM-ACACCATGGCACAAGTGAGA-TAMRA | chr12:63,935,220  and chr12:64,130,260 | 60 |
| 9) PRDM9 ZF PCR  PN0.6F  PN2.5R | TGAGGTTACCTAGTCTGGCA  ATAAGGGGTCAGCAGACTTC |  | 55 |
| 10) PRDM9 ZF sequencing primers  PN1.2F  PN2.4R | TGAATCCAGGGAACACAGGC  GCAAGTGTGTGGTGACCACA |  | 55 |
