# Supplementary material for: Fine Characterisation of a Recombination Hotspot at the DPY19L2 Locus and Resolution of the Paradoxical Excess of Duplications over Deletions in the General Population
Source: PLoS Genet. 2013 Mar 21;9(3):e1003363. doi: 10.1371/journal.pgen.1003363 (PMC3605140; doi:10.1371/journal.pgen.1003363)
Supplement: Table S2 — Sequence of the recombination hotspot region of control subjects (10 Europeans and 10 North Africans) for the identification of LCR-specific markers and determination of the precise localisation of the breakpoints of 15 globozoospermia patients. (DOC) [file pgen.1003363.s003.doc]

Table S2: Number (Nb) and percentage (%) of deleted (del.) versus duplicated (dup.) allele. Total number of recombinant alleles (deleted + duplicated : total recomb.) and frequency of the different alleles in the studied populations

|  | Nb of individuals | Nb del. (%) | Freq. of del. | Nb dup. (%) | Freq. of dup. | Total recomb | Freq. of recomb. |
| --- | --- | --- | --- | --- | --- | --- | --- |
| Total DGV | 6575 | 26 (24) | 0,40 | 83 (76) | 1,26 | 109 | 1,66 |
| Home CGH cohort + PCR | 1999 | 4 (19) | 0,18 | 17 (81) | 0,88 | 21 | 1,06 |
| Total | 8574 | 30 (23) | 0,35 | 100 (77) | 1,18 | 130 | 1,53 |
