# Supplementary material for: Fine Characterisation of a Recombination Hotspot at the DPY19L2 Locus and Resolution of the Paradoxical Excess of Duplications over Deletions in the General Population
Source: PLoS Genet. 2013 Mar 21;9(3):e1003363. doi: 10.1371/journal.pgen.1003363 (PMC3605140; doi:10.1371/journal.pgen.1003363)
Supplement: Table S3 — Number and percentage (%) of recombined alleles with breakpoints located within (inside) or outside of the LCR. (DOC) [file pgen.1003363.s004.doc]

Table S3 : Number and percentage (%) of recombined alleles with breakpoints located within (Inside) or outside of the LCR.

|  | Inside LCR | |  | Outside LCR | |  | Total Inside LCR | Total Outside LCR | Total recomb |
| --- | --- | --- | --- | --- | --- | --- | --- | --- | --- |
|  | del | dup |  | del | dup |  |  |  |  |
| Total DGV | 22 (85) | 61 (73) |  | 4 (15) | 22 (27) |  | 83 (76) | 26 (24) | 109 |
| Home CGH cohort + PCR | 4 (100) | 15 (88) |  | 0 | 2 (12) |  | 19 (90) | 2 (10) | 21 |
| Total | 26 (87) | 76 (76) |  | 4 (13) | 24 (24) |  | 102 (78) | 28 (22) | 130 |
